# The impact of the COVID-19 pandemic on persistence in the European stock markets^☆^

**DOI:** 10.1016/j.heliyon.2022.e11560

**Published:** 2022-11-12

**Authors:** Guglielmo Maria Caporale, Luis Gil-Alana, Isabel Arrese Lasaosa

**Affiliations:** a Brunel University London, United Kingdom; b University of Navarra, Pamplona, Spain

**Keywords:** Covid-19 pandemic, European stock market indices, Persistence, Fractional integration

## Abstract

This paper analyses the impact of the Covid-19 pandemic on the degree of persistence of European stock markets. Specifically, it uses fractional integration methods to estimate persistence at the daily, weekly and monthly frequencies in the case of ten major European stock market indices; the effects of the pandemic are assessed by comparing the pre-pandemic estimates (over the period 2005–2019) to those from a sample extended until July 2021 which includes the pandemic period. The approach used is more general than the standard one based on the stationarity versus non-stationarity dichotomy and allows for a wider range of dynamic processes. Three different model specifications are considered, and these are estimated under two alternative assumptions for the disturbances (white noise and autocorrelation). The findings indicate that there has not been any significant impact of the Covid-19 pandemic on the degree of persistence of the European stock market indices, though their volatility persistence has decreased.

## 1 Introduction

The Covid-19 pandemic has been one of the greatest challenges faced by the world economy, including the European Union (Consilium, 2021 - see Caporale and Cerrato, 2020, for the initial policy responses in Europe). It has had significant effects both on the real economy and on financial markets. For instance, Shehzad et al. (2020) provided evidence that the European and US stock markets have reacted more strongly than Asian ones – in particular, the S&P 500 index has dropped by 30% since the start of the pandemic. Corbet et al. (2020) reported an increase in the correlation between the volatility of the Chinese stock market and of Bitcoin at the peak of the pandemic. Ali et al. (2020) found higher stock market volatility during the pandemic in the US, the UK, Germany, and South Korea, which reflects the higher uncertainty faced by investors; this is lower in high-trust compared to low-trust countries (Engelhardt et al., 2021).

This paper focuses on the impact of the Covid-19 pandemic on the degree of persistence of various European stock market indices (DAX, FTSE100, CAC40, FTSE MIB, IBEX35, AEX, SMI, BIST100, WIG20, OMXS30) as well as their volatility. For this purpose fractional integration methods are applied to compare the period from January 2005 to December 2019, namely before the pandemic, to that until July 2021, the latter including the pandemic. The econometric approach used for the analysis is more general than the standard one adopted by many previous studies; in particular, it is not restricted by the classical dichotomy between I(0) and I(1) processes and instead it allows for a much wider range of possible stochastic behaviours, including, for example, the case of nonstationary but nevertheless mean-reverting processes occurring when the order of integration is in the range [0.5, 1). Although it has previously been used in some other papers examining the effects of the Covid-19 pandemic on stock markets (see, e.g., Abakah et al., 2021, 2022; Caporale et al., 2022a; 2022c), none of the previous studies has provided comprehensive evidence for Europe based on this approach as in the case of the present study, this being its main contribution to the literature.

The structure of paper is as follows: Section 2 briefly reviews the existing literature to put the analysis in context; Section 3 outlines the methodology used; Section 4 describes the data and the empirical results; Section 5 concludes.

## 2 Literature review

Following the onset of the Covid-19 pandemic, several studies were carried out to examine specifically its impact on stock markets. For instance, Ramelli and Wagner (2020) provided some initial evidence of a drop in stock prices reflecting concerns about future economic prospects. The role of bigger uncertainty was also analysed by Baker et al. (2020), who pointed out that in March 2020 the VIX (Chicago Board Options Exchange's Volatility Index) peaked at a higher level than during the Great Recession. Zhang et al. (2020) found a substantial increase in risk in global financial markets using correlation analysis, graph theory and a minimum spanning tree (MST) approach. Higher stock market volatility could also be the result of rapidly changing attitudes towards risk or investor sentiment (see Cox et al., 2020; Gormsen and Koijen, 2020).

Al-Awadhi et al. (2020) used panel data methods to investigate the effects of the Covid-19 pandemic on Chinese stock returns and found a significant impact total confirmed cases and total deaths on both the Hang Seng Index and Shanghai Stock Exchange Composite Index. Also in a panel context, Salisu and Vo (2020) found that an increase in health news searches leads to lower stock returns. Interestingly, stock returns appeared to have a high predictive content for future economic activity (Davis et al., 2021); besides, there is evidence of a strong linkage between the geographical spread of Covid-19 and the degree of financial instability (Albulescu, 2020). Finally, Štifanić et al. (2020) used very different methods, namely the stationary wavelet transform (SWT) and bidirectional long short-term memory (BDLSTM) networks, and concluded that the Covid-19 pandemic caused only a temporary slump in crude oil and US stock prices.

Caporale et al. (2022b) analysed the impact of the Covid-19 pandemic on both stock market returns and their volatility in the G20 countries using a dynamic panel data model. Their framework takes into account the epidemiological situation (using a Covid-19 index based on a Balanced Worth (BW) methodology) as well as the restrictive measures and fiscal and monetary responses adopted by national governments. They found that restrictions and other policy measures played a more important role in the G7 countries than the Covid-19 pandemic in driving both returns and their volatility, whilst the health crisis itself was the main factor affecting stock markets in the case the non-G7 stock markets.

A few recent papers apply fractional integration methods to assess the impact of the Covid-19 pandemic. For instance, Abakah et al. (2021) analysed the effects of containment measures and monetary and fiscal responses on the daily S&P500, the US Treasury Bond Index (USTB), the S&P Green Bond Index (GREEN) and the Dow Jones (DJ) Islamic World Market Index (ISLAM) over the period 1/01/2020-10/03/2021. They provided evidence of the positive impact of (monetary) policy announcements as well as changes in the effective Federal funds rate. Caporale et al. (2022a) examined instead the possible effects of the Covid-19 pandemic on the degree of persistence of US monthly stock prices and bond yields. Bond yields are generally found to be more persistent; moreover, the recursive analysis shows no impact of the Covid-19 pandemic on the persistence of stock prices, whilst there is an increase in the case of both 10- and 1- year bond yields but not of their spread. Caporale et al. (2022c) focused instead on the impact of US policy responses (containment and health measures, income support policy, debt-relief policy, changes in the Effective Federal Funds Rate, monetary and fiscal announcements) to the Covid-19 pandemic on US sectoral stock indices. They found that changes in the Effect Federal Funds Rate had the most significant (positive) effect also at the sectoral level in most cases, whilst the mortality rate affected negatively most sectoral stock indices. Finally, Abakah et al. (2022) investigated the response to the pandemic and policy measures of various cryptocurrencies as well as technology stocks. Their findings imply that fiscal measures had positive effects whilst again the mortality rate had a negative impact.

Various other contributions have also been published in Heliyon Business and Economics, where there is an ongoing debate on the impact of the Covid-19 pandemic on stock markets. For instance, Fassas (2020) analysed the dynamic connectedness of the risk premium in international developed and emerging equity markets and concluded that it was strengthened by the pandemic. Kamaludina et al. (2021) focused on the ASEAN-5 countries and using wavelet analysis found different degrees of coherency between those stock markets at different stages of the pandemic. Kapalu and Kodongo (2022) investigated the effects of Covid-19 on bond yields and stock returns and established that these differed across countries depending on the policy response; in particular, in countries with fewer restrictions investor fear led to higher bond yields. Oanh (2022) analyzed the impact of Covid-19 vaccination on the stock markets of 77 countries using a panel data vector autoregression (PVAR) model and interestingly found that it was positive on those of developing countries and negative on those of developed countries, where therefore additional measures such as mask wearing appear to be necessary. Finally, Bossmana et al. (2022) found that the volatility spillovers across and within Islamic and/or G7 markets are time-varying and frequency-dependent and that conventional stocks are more volatile; moreover, during turbulent periods such as the Covid-19 pandemic contagion increases.

The studies reviewed above use a variety of empirical methods, both in a time series and in a panel context (and also use wavelets). As previously emphasised, the vast majority of them assume that the differencing parameter characterising the series of interest is an integer, which is a very restrictive assumption concerning their stochastic behaviour. There are only a few studies (see, e.g., Abakah et al., 2021, 2022; Caporale et al., 2022a; 2022c) allowing this parameter also to take fractional values and thus incorporating a wide spectrum of stochastic processes. However, all of them focus on the US markets. By contrast, the present one uses fractional integration methods to consider the possible impact of the Covid-19 pandemic on a number of European stock markets, an issue which to our knowledge has not been investigated before applying this framework. Therefore the obtained evidence is novel as well as informative and represents an original contribution to the literature in general and specifically to the debate on these issues being conducted in Heliyon Business and Economics, since none of the studies published there has a European focus and none provides evidence on persistence based on our more general approach.

## 3 Methodology

The measure of persistence used in this paper is the estimated fractional integration parameter from an appropriately specified model. Alternative measures, such as the AR(1) coefficient or the sum of the AR(p) coefficients of the process under examination, are questionable if their values are close to the unit circle as often in practice. Fractional integration is a more general case and does not produce an abrupt change in the behaviour of the series around the unit root.

More specifically, a stochastic process is said to be integrated of order d, denoted by I(d), if it can be represented as:

(1) $${\left(1-L\right)}^{d}x_{t}=u_{t},t=1\;,2\;,...,$$

where L is the lag-operator ($Lx_{t}=x_{t-1}$): *d* can be any real value, and *u*_*t*_ is an *I(0)* process which is covariance stationary and has a spectral density function that is positive and finite at any frequency. The category of I(0) processes includes the white noise case but also a wide range of specifications such as the stationary autoregressive moving average (ARMA) class of models.

The polynomial appearing on the left-hand side in Eq. (1) can be defined in terms of its Binomial expansion, such that, for all real *d*,

(2) $${\left(1-L\right)}^{d}=\sum_{j=0}^{\infty }\frac{\Gamma \left(j-d\right)}{\Gamma \left(j+1\right)\;\Gamma \left(-d\right)}\;L^{j}\text{,}$$

where *Γ(x)* is the Gamma function.

When *d = 0* in Eq. (1), *x*_*t*_ *= u*_*t*_*,* and therefore *x*_*t*_ is *I(0)*, and possibly “*weakly autocorrelated*” (also known as “*weakly dependent*”), with the autocorrelations decaying exponentially if the underlying disturbances are autoregressive. If *0 < d < 0.5*, *x*_*t*_ is still covariance stationary, but its lag-u autocovariance *γ*_*u*_ decreases very slowly, in fact hyperbolically, according to Eq. (2), and therefore the *γ*_*u*_ are absolutely non-summable. In that case *x*_*t*_ is said to exhibit long memory given that its spectral density *f(λ)* is unbounded at the origin (see Eq. (3)). Finally, it is important to note that as d in (1) increases beyond 0.5 and towards 1 (the unit root case), the variance of the partial sums of *x*_*t*_ increases in magnitude. This is also true for *d > 1*, so a large class of nonstationary processes may be described by (1) with *d ≥ 0.5*.^1^

As mentioned before, the main advantage of the fractional integration framework is its generality that allows to consider a wide range of model specifications, including anti-persistence (d < 0), short memory (d = 0), stationary long memory (0 < d <0.5); nonstationary mean reverting processes (0.5 ≤ d < 1); unit roots (d = 1) or even explosive processes (d > 1).

The method employed in this paper to estimate the fractional differencing parameter *d* is based on the Whittle function (an approximation to the likelihood function) expressed in the frequency domain (Dahlhaus, 1989) and uses a testing approach developed in Robinson (1994) and widely applied (Gil-Alana and Robinson, 1997; Gil-Alana and Moreno, 2012; Abbritti et al., 2016). The use of alternative long memory methodologies produced almost identical results to those reported in the paper (these are available from the authors upon request).

## 4 Data description and empirical results

**Figure 1:**
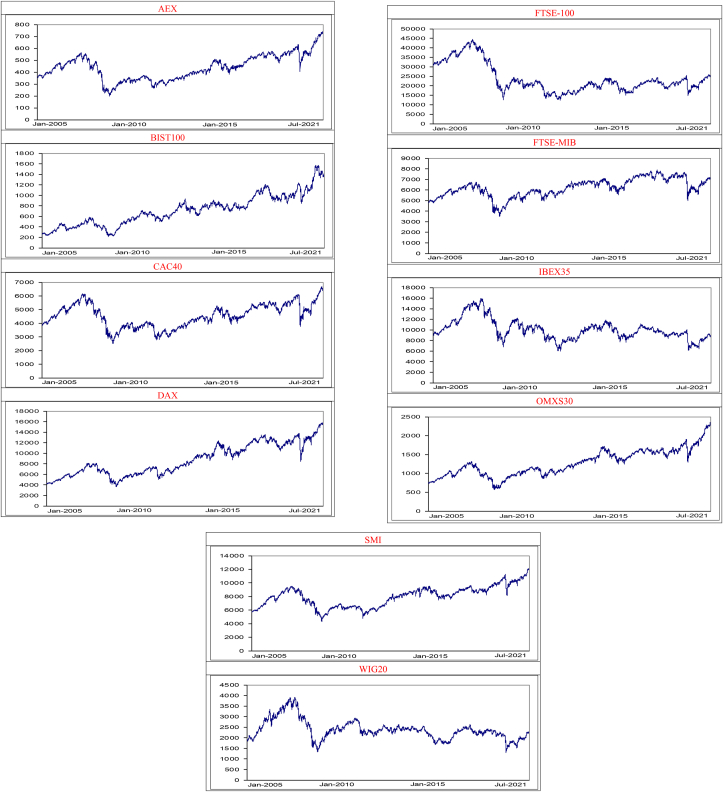
Time series plots of the daily series. Note: AEX: Amsterdam Exchange Index, BIST100: Borsa Istanbul stock exchange, CAC40: Cotation Assistée en Continu, DAX: Deutscher Aktienindex, FTSE100: Financial Times Stock Exchange 100 Index, FTSEMIB: Milano Indice di Borsa, IBEX35: Índice Bursátil Español, OMXS30: Options Market Index Stockholm, SMI: Swiss Market Index, WIG20: Warszawski Indeks Giełdowy.

We examine the behaviour of ten European stock market indices, namely AEX (Amsterdam Exchange Index, the Netherlands), BIST100 (Borsa Istanbul stock exchange, Turkey), CAC40 (Cotation Assistée en Continu, France) DAX (Deutscher Aktienindex, Germany), FTSE100 (Financial Times Stock Exchange 100 Index, UK), FTSEMIB (Milano Indice di Borsa, Italy), IBEX35 (Índice Bursátil Español, Spain), OMXS30, Options Market Index Stockholm, Sweden), SMI (Swiss Market Index, Switzerland), WIG20 (Warszawski Indeks Giełdowy, Poland). Specifically, we consider the daily, weekly, and monthly closing prices over the period going from January 2005 to July 2021;^2^ the data source is Thomson Reuters Eikon. In order to analyse the possible impact of the Covid-19 parameter on the fractional integration parameter d, which is a measure of persistence, first we estimate the models from January 2005 to December 2019, then we re-estimate them for the full sample up to July 2021. Figure 1 displays plots of the daily series (the weekly and monthly ones look very similar), whilst Table 1a,1b,1c report some descriptive statistics for each frequency.

**Table 1a:** Descriptive statistics: Daily data.

Series	Max. value	Min value	Mean	Std. Dev-	J-B stat.
AEX	740.69	199.25	438.57	105.11	55.51
BIST100	1570.42	212.2827	712.82	293.05	155.93
CAC40	6666.26	2519.29	4552.2	845.49	97.27
DAX	15790.51	3666.41	8761.19	2972.31	259.82
FTSE-100	7877.45	3512.09	6193.09	858.46	106.95
FTSE-MIB	44364	12362.51	23592.09	7503.82	860.26
IBEX35	15945.7	5956.3	10052.03	1927.14	550.6
OMXS30	2349.53	567.61	1274.02	345.95	113.14
SMI	12085.51	4307.67	7984.45	1515.04	68.88
WIG20	3910	1305.73	2375.31	469.4	725.71

**Table 1b:** Descriptive statistics: Weekly data.

Series	Max. value	Min value	Mean	Std. Dev-	J-B stat.
AEX	740.7	199.5	438.80	105.67	12.45
BIST100	1560.34	219.6596	714.25	292.55	33.38
CAC40	6622.87	2534.45	4555.09	850.35	20.88
DAX	15785.23	3666.41	8778.97	2975.49	56.23
FTSE-100	7778.79	3530.73	6200.04	861.28	24.42
FTSE-MIB	44364	12739.98	23548.27	7488.01	199.85
IBEX35	15823.7	6065	10047.21	1931.7	127.98
OMXS30	2351.848	567.613	1275.62	346.70	26
SMI	12047.86	4311.61	7988.96	1517.09	14.54
WIG20	3899.59	1365.97	2373.63	469.21	165.61

**Table 1c:** Descriptive statistics: Monthly data.

Series	Max. value	Min value	Mean	Std. Dev-	J-B stat.
AEX	15795.95	3843.74	439.93	2971.96	2.55
BIST100	1476.72	235.9164	716.99	291.28	7.05
CAC40	6550.52	2702.48	4567.52	848.38	4.72
DAX	15795.95	3843.74	8810.70	2971.96	12.5
FTSE-100	7748.76	3830.09	6198.92	848.49	5.21
FTSE-MIB	43755	12873.84	23544.06	7489.72	48.45
IBEX35	15890.5	6089.8	10047.59	1941.79	29.61
OMXS30	2351.73	617.376	1280.57	349.21	6.16
SMI	12056.86	4690.67	8008.49	1517.6	3.15
WIG20	3877.62	1372.47	2375.51	470.14	36.49

The estimated model is the following:

(3) $$y_{t}=\alpha +\beta \;t+x_{t}\;,{\left(1-L\right)}^{d_{o}}x_{t}=u_{t},t=1\;,2\;,...\;\text{,}$$

where y_t_ is the observed time series, α and β are the intercept and the time trend coefficient respectively, and d is the differencing parameter.

We start by presenting the results based on the sample ending on 31 December 2019, that is, just before the Covid-19 pandemic (Tables 2, 3, 4, 5, 6, 7). Tables 2, 4, and 6 display the estimated values of d (and the 95% confidence bands of the non-rejection values of d using Robinson's (1994) tests) for the daily, weekly and monthly series respectively and three model specifications: i) no deterministic terms, ii) an intercept, and iii) an intercept as well as a linear time trend. The full set of estimates for the three frequencies considered is reported in Tables 3, 5, and 7; those from the selected model in each case (on the basis of the statistical significance of the estimated coefficients) are shown in bold. The upper and lower half of the tables report the results for the case of white noise and autocorrelated errors in (3) respectively; in the latter case we use the exponential spectral model of Bloomfield (1973), which is an approximation of AR structures in the frequency domain.

**Table 2:** Estimates of the differencing parameter. Daily data. Sample ending on 31/12/2019.

Series (- 31/12/2019)	d (95% band)	Constant	A constant and a linear time trend
i) No autocorrelation			
AEX	1.00 (0.97, 1.03)	**0.99 (0.96, 1.01)**	0.99 (0.96, 1.01)
BIST100	1.00 (0.98, 1.03)	**1.01 (0.98, 1.04)**	1.01 (0.98, 1.04)
CAC40	1.00 (0.97, 1.03)	**0.95 (0.92, 0.97)**	0.95 (0.92, 0.97)
DAX	1.00 (0.97, 1.03)	0.98 (0.96, 1.01)	**0.98 (0.96, 1.01)**
FTSE-100	1.00 (0.97, 1.02)	**0.94 (0.92, 0.97)**	0.94 (0.92, 0.97)
FTSE-MIB	1.00 (0.97, 1.03)	**0.97 (0.94, 0.99)**	0.97 (0.94, 0.99)
IBEX35	1.00 (0.97, 1.03)	**0.97 (0.94, 1.00)**	0.97 (0.94, 1.00)
OMXS30	1.00 (0.97, 1.03)	0.94 (0.91, 0.97)	**0.94 (0.91, 0.97)**
SMI	1.00 (0.97, 1.03)	**0.98 (0.95, 1.01)**	0.98 (0.95, 1.01)
WIG20	1.00 (0.97, 1.02)	**1.00 (0.97, 1.02)**	1.00 (0.97, 1.02)
ii) With autocorrelation			
AEX	0.99 (0.95, 1.04)	**0.98 (0.94, 1.03)**	0.98 (0.94, 1.03)
BIST100	0.99 (0.95, 1.04)	**1.02 (0.98, 1.07)**	1.02 (0.98, 1.07)
CAC40	1.00 (0.96, 1.05)	**0.95 (0.92, 1.00)**	0.95 (0.92, 1.00)
DAX	1.00 (0.96, 1.04)	**0.98 (0.92, 1.03)**	0.98 (0.92, 1.03)
FTSE-100	0.99 (0.95, 1.03)	**0.94 (0.89, 0.98)**	0.94 (0.89, 0.98)
FTSE-MIB	1.00 (0.96, 1.05)	**1.00 (0.96, 1.05)**	1.00 (0.96, 1.05)
IBEX35	1.00 (0.95, 1.04)	**0.95 (0.91, 1.01)**	0.95 (0.91, 1.00)
OMXS30	1.00 (0.96, 1.04)	0.92 (0.88, 0.97)	**0.92 (0.88, 0.97)**
SMI	1.00 (0.96, 1.04)	0.89 (0.85, 0.93)	**0.89 (0.85, 0.93)**
WIG20	1.00 (0.96, 1.04)	**0.95 (0.91, 1.00)**	0.95 (0.91, 1.00)

Note: in parenthesis, the 95% confidence intervals of the non-rejection values of d. In bold the estimated values from the selected model specification.

**Table 3:** Estimated coefficients of the selected models. Daily data. Sample ending on 31/12/2019.

Series (- 31/12/2019)	No terms	Constant (t-value)	Time trend (t-value)
i) No autocorrelation			
AEX	0.99 (0.96, 1.01)	5.865 (450.43)	---
BIST100	1.01 (0.98, 1.04)	5.523 (323.83)	---
CAC40	0.95 (0.92, 0.97)	8.259 (597.39)	---
DAX	0.98 (0.96, 1.01)	8.363 (618.66)	0.00036 (1.87)
FTSE-100	0.94 (0.92, 0.97)	8.406 (738.61)	---
FTSE-MIB	0.97 (0.94, 0.99)	10.344 (649.77)	---
IBEX35	0.97 (0.94, 1.00)	9.128 (611.29)	---
OMXS30	0.94 (0.91, 0.97) 0.94 (0.91, 0.97)	6.624 (485.48)	0.00024 (1.70)
SMI	0.98 (0.95, 1.01)	8.661 (781.43)	---
WIG20	1.00 (0.97, 1.02)	7.578 (519.15)	---
ii) With autocorrelation			
AEX	0.98 (0.94, 1.03)	6.006 (429.48)	
BIST100	1.02 (0.98, 1.07)	5.980 (366.95)	**---**
CAC40	0.95 (0.92, 1.00)	8.383 (557.17)	**---**
DAX	0.98 (0.92, 1.03)	8.751 (591.81)	**---**
FTSE-100	0.94 (0.89, 0.98)	8.617 (692.25)	**---**
FTSE-MIB	1.00 (0.96, 1.05)	10.233 (577.55)	**---**
IBEX35	0.95 (0.91, 1.01)	9.348 (572.27)	**---**
OMXS30	0.92 (0.88, 0.97)	6.759 (478.56)	0.00033 (2.38)
SMI	0.89 (0.85, 0.93)	8.865 (772.16)	0.00018 (2.01)
WIG20	0.95 (0.91, 1.00)	7.829 (523.57)	**---**

**Note:** the values in column 2 refers are the estimates of d and their 95% confidence band. In parenthesis, in column 3 and 4, the t-values of the deterministic terms.

**Table 4:** Estimates of the differencing parameter. Weekly data. Sample ending on 31/12/2019.

Series: (- 31/12/2019)	d (95% band)	Constant	A constant and a linear time trend
i) No autocorrelation			
AEX	1.00 (0.95, 1.05)	**0.99 (0.94, 1.04)**	0.99 (0.94, 1.04)
BIST100	1.01 (0.96, 1.06)	**1.00 (0.95, 1.05)**	1.00 (0.95, 1.05)
CAC40	1.00 (0.95, 1.05)	**0.93 (0.88, 0.98)**	0.93 (0.89, 0.98)
DAX	0.99 (0.95, 1.04)	0.94 (0.90, 1.00)	**0.95 (0.90, 1.00)**
FTSE-100	1.00 (0.95, 1.05)	**0.91 (0.86, 0.96)**	0.91 (0.86, 0.96)
FTSE-MIB	1.00 (0.95, 1.05)	**0.99 (0.94, 1.04)**	0.99 (0.94, 1.04)
IBEX35	1.00 (0.95, 1.05)	**0.93 (0.87, 0.98)**	0.93 (0.87, 0.98)
OMXS30	1.00 (0.95, 1.05)	**0.94 (0.89, 0.99)**	0.94 (0.89, 0.99)
SMI	1.00 (0.95, 1.05)	**0.90 (0.86, 0.94)**	0.90 (0.86, 0.94)
WIG20	1.00 (0.95, 1.05)	**1.01 (0.96, 1.07)**	1.01 (0.96, 1.07)
ii) With autocorrelation			
AEX	0.99 (0.92, 1.09)	**1.03 (0.95, 1.11)**	1.03 (0.95, 1.11)
BIST100	0.99 (0.92, 1.09)	**1.06 (0.97, 1.16)**	1.06 (0.97, 1.16)
CAC40	0.99 (0.92, 1.08)	**0.98 (0.91, 1.07)**	0.98 (0.91, 1.07)
DAX	0.98 (0.91, 1.08)	**0.99 (0.91, 1.09)**	0.99 (0.91, 1.09)
FTSE-100	0.99 (0.92, 1.09)	**0.93 (0.85, 1.03)**	0.93 (0.85, 1.03)
FTSE-MIB	0.99 (0.92, 1.09)	**1.02 (0.94, 1.13)**	1.02 (0.94, 1.13)
IBEX35	0.99 (0.92, 1.09)	**1.01 (0.93, 1.11)**	1.01 (0.93, 1.11)
OMXS30	0.99 (0.92, 1.09)	**1.00 (0.92, 1.08)**	1.00 (0.92, 1.08)
SMI	0.99 (0.92, 1.08)	**1.04 (0.97, 1.12)**	1.04 (0.97, 1.12)
WIG20	0.99 (0.92, 1.08)	**0.98 (0.91, 1.05)**	0.98 (0.91, 1.05)

**Note:** in parenthesis, the 95% confidence intervals of the non-rejection values of d. In bold the estimated values from the selected model specification.

**Table 5:** Estimated coefficients of the selected models. Weekly data. Sample ending on 31/12/2019.

Series (- 31/12/2019)	No terms	Constant (t-value)	Time trend (t-value)
i) No autocorrelation			
AEX	0.99 (0.94, 1.04)	5.870 (211.11)	---
BIST100	1.00 (0.95, 1.05)	5.533 (154.69)	---
CAC40	0.93 (0.88, 0.98)	8.264 (293.54)	---
DAX	0.95 (0.90, 1.00)	8.368 (288.25)	0.0014 (1.87)
FTSE-100	0.91 (0.86, 0.96)	8.408 (364.35)	---
FTSE-MIB	0.99 (0.94, 1.04)	10.345 (320.98)	---
IBEX35	0.93 (0.87, 0.98)	9.121 (297.09)	---
OMXS30	0.94 (0.89, 0.99)	6.618 (242.73)	---
SMI	0.90 (0.86, 0.94)	8.657 (365.41)	---
WIG20	1.01 (0.96, 1.07)	7.551 (255.03)	---
ii) With autocorrelation			
AEX	1.03 (0.95, 1.11)	5.870 (211.31)	---
BIST100	1.06 (0.97, 1.16)	5.530 (155.24)	---
CAC40	0.98 (0.91, 1.07)	8.263 (297.25)	---
DAX	0.99 (0.91, 1.09)	8.370 (288.99)	---
FTSE-100	0.93 (0.85, 1.03)	8.488 (363.27)	---
FTSE-MIB	1.02 (0.94, 1.13)	10.345 (321.25)	---
IBEX35	1.01 (0.93, 1.11)	9.109 (297.57)	---
OMXS30	1.00 (0.92, 1.08)	6.617 (242.46)	---
SMI	1.04 (0.97, 1.12)	8.653 (365.91)	---
WIG20	0.98 (0.91, 1.05)	7.551 (255.31)	---

**Note:** the values in column 2 refers to the estimates of d and 95% confidence band. In parenthesis, in column 3 and 4, the t-values of the deterministic terms.

**Table 6:** Estimates of the differencing parameter. Monthly data. Sample ending on 31/12/2019.

Series (- 31/12/2019)	d (95% band)	Constant	A constant and a linear time trend
i) No autocorrelation			
AEX	0.99 (0.91, 1.11)	**1.09 (0.99, 1.21)**	1.09 (0.99, 1.21)
BIST100	0.99 (0.89, 1.11)	**0.98 (0.87, 1.12)**	0.98 (0.87, 1.12)
CAC40	0.99 (0.90, 1.11)	**1.05 (0.95, 1.18)**	1.05 (0.95, 1.18)
DAX	0.98 (0.89, 1.11)	**1.04 (0.92, 1.18)**	1.04 (0.93, 1.18)
FTSE-100	0.98 (0.89, 1.11)	**0.97 (0.87, 1.09)**	0.97 (0.87, 1.09)
FTSE-MIB	0.99 (0.89, 1.11)	**1.05 (0.95, 1.17)**	1.05 (0.95, 1.17)
IBEX35	0.98 (0.89, 1.11)	**1.02 (0.92, 1.15)**	1.02 (0.92, 1.15)
OMXS30	0.99 (0.90, 1.11)	**1.05 (0.95, 1.17)**	1.05 (0.95, 1.17)
SMI	0.99 (0.89, 1.11)	**1.13 (1.04, 1.26)**	1.13 (1.04, 1.25)
WIG20	0.99 (0.90, 1.11)	**1.02 (0.93, 1.14)**	1.02 (0.93, 1.14)
ii) With autocorrelation			
AEX	0.97 (0.81, 1.16)	**1.04 (0.86, 1.29)**	1.04 (0.86, 1.29)
BIST100	0.94 (0.80, 1.16)	0.94 (0.70, 1.26)	**0.95 (0.73, 1.27)**
CAC40	0.96 (0.82, 1.18)	**0.94 (0.79, 1.15)**	0.94 (0.80, 1.15)
DAX	0.95 (0.81, 1.17)	0.83 (0.66, 1.06)	**0.85 (0.66, 1.06)**
FTSE-100	0.95 (0.81, 1.17)	**0.96 (0.79, 1.21)**	0.97 (0.78, 1.21)
FTSE-MIB	0.96 (0.82, 1.18)	**0.98 (0.81, 1.19)**	0.98 (0.82, 1.19)
IBEX35	0.95 (0.82, 1.17)	**0.93 (0.76, 1.17)**	0.93 (0.75, 1.17)
OMXS30	0.96 (0.80, 1.18)	**1.06 (0.84, 1.34)**	1.06 (0.86, 1.33)
SMI	0.96 (0.82, 1.18)	**1.09 (0.92, 1.32)**	1.09 (0.93, 1.32)
WIG20	0.94 (0.81, 1.15)	**1.04 (0.86, 1.28)**	1.05 (0.86, 1.30)

**Note:** in parenthesis, the 95% confidence intervals of the non-rejection values of d. In bold the estimated values from the selected model specification.

**Table 7:** Estimated coefficients of the selected models. Monthly data. Sample ending on 31/12/2019.

Series (- 31/12/2019)	No terms	Constant (t-value)	Time trend (t-value)
i) No autocorrelation			
AEX	1.09 (0.99, 1.21)	5.883 (121.71)	---
BIST100	0.98 (0.87, 1.12)	5.611 (74.81)	---
CAC40	1.05 (0.95, 1.18)	8.269 (179.57)	---
DAX	1.04 (0.92, 1.18)	8.353 (165.09)	---
FTSE-100	0.97 (0.87, 1.09)	8.488 (229.90)	---
FTSE-MIB	1.05 (0.95, 1.17)	10.351 (176.77)	---
IBEX35	1.02 (0.92, 1.15)	9.128 (169.30)	---
OMXS30	1.05 (0.95, 1.17)	6.605 (143.74)	---
SMI	1.13 (1.04, 1.26)	8.654 (250.35)	---
WIG20	1.02 (0.93, 1.14)	7.540 (128.00)	---
ii) With autocorrelation			
AEX	1.04 (0.86, 1.29)	5.835 (121.27)	---
BIST100	0.95 (0.73, 1.27)	5.603 (74.60)	0.0078 (1.78)
CAC40	0.94 (0.79, 1.15)	8.276 (180.66)	---
DAX	0.85 (0.66, 1.06)	8.358 (169.56)	0.0061 (3.35) (4.39)
FTSE-100	0.96 (0.79, 1.21)	8.488 (229.91)	---
FTSE-MIB	0.98 (0.81, 1.19)	10.352 (176.82)	---
IBEX35	0.93 (0.76, 1.17)	9.133 (170.41)	---
OMXS30	1.06 (0.84, 1.34)	6.604 (143.82)	---
SMI	1.09 (0.92, 1.32)	8.656 (248.58)	---
WIG20	1.04 (0.86, 1.28)	7.537 (128.19)	---

**Note:** the values in column 2 are the estimates of d and their 95% confidence bands. In parenthesis, in column 3 and 4, the t-values of the deterministic terms.

For the daily series (Tables 2 and 3) a time trend is required in two cases with white noise errors (DAX and OMXS-30), and also for OMXS-30 and SMI with weak autocorrelation. In all cases the coefficients are significantly positive.

As for the differencing parameter, evidence of mean reversion (namely of d < 1) is found in a number of cases: CAC-40, FTSE-MIB, FTSE-100 and OMXS-30 with white noise errors, and in the last two along with SMI with autocorrelation. In the remaining cases, under the assumption of white noise errors the unit root null hypothesis (i.e., d = 1) cannot be rejected, which is consistent with the Efficient Market Hypothesis (EMH), at least in its weak form.

Concerning the weekly series (Tables 4 and 5), a significant positive time trend is found only in the case of the DAX with white noise errors, and mean reversion takes place in half of the cases with white noise errors (CAC, FTSE-100, IBEX, OMXS and SMI) but not in a single case with autocorrelation. Finally, for the monthly series the time trend is only significant in the case of BIST-100 and DAX with autocorrelated errors, and mean reversion is not found in any single case.

Next we re-estimate the models over the full sample until July 2021 to assess the impact of the Covid-19 pandemic on persistence. Tables 8, 9, 10, 11, 12, and 13 report these results: Tables 8 and 9 concern the daily data, Tables 10 and 11 the weekly data, and Tables 12 and 13 the monthly ones.

**Table 8:** Estimates of the differencing parameter. Daily data. Sample ending on 13 July 2021.

Series (-13/07/2021)	d (95% band)	Constant	A constant and a linear time trend
i) No autocorrelation			
AEX	1.00 (0.97, 1.03)	**0.99 (0.97, 1.02)**	0.99 (0.97, 1.02)
BIST100	1.00 (0.97, 1.02)	**1.01 (0.99, 1.04)**	1.01 (0.99, 1.04)
CAC40	1.00 (0.97, 1.02)	**0.96 (0.94, 0.99)**	0.96 (0.93, 0.99)
DAX	1.00 (0.97, 1.03)	0.99 (0.97, 1.02)	**0.99 (0.97, 1.02)**
FTSE-100	1.00 (0.97, 1.02)	**0.95 (0.93, 0.98)**	0.95 (0.93, 0.98)
FTSE-MIB	1.00 (0.97, 1.03)	**0.97 (0.95, 1.00)**	0.97 (0.95, 1.00)
IBEX35	1.00 (0.97, 1.02)	**0.98 (0.95, 1.01)**	0.98 (0.95, 1.01)
OMXS30	1.00 (0.97, 1.02)	0.94 (0.92, 0.97)	**0.94 (0.92, 0.97)**
SMI	1.00 (0.97, 1.02)	**0.98 (0.95, 1.00)**	0.98 (0.95, 1.00)
WIG20	1.00 (0.97, 1.02)	**1.00 (0.98, 1.03)**	1.00 (0.98, 1.03)
ii) With autocorrelation			
AEX	0.99 (0.95, 1.04)	**0.98 (0.94, 1.03)**	0.98 (0.94, 1.03)
BIST100	0.99 (0.95, 1.04)	**1.02 (0.98, 1.07)**	1.02 (0.98, 1.07)
CAC40	1.00 (0.96, 1.05)	**0.95 (0.91, 1.00)**	0.95 (0.91, 1.00)
DAX	1.00 (0.96, 1.04)	**0.98 (0.94, 1.03)**	0.98 (0.94, 1.03)
FTSE-100	0.99 (0.95, 1.04)	**0.94 (0.89, 0.98)**	0.94 (0.89, 0.98)
FTSE-MIB	1.00 (0.96, 1.05)	**1.00 (0.96, 1.05)**	1.00 (0.96, 1.05)
IBEX35	1.00 (0.95, 1.05)	**0.95 (0.91, 1.00)**	0.95 (0.91, 1.00)
OMXS30	1.00 (0.96, 1.05)	0.92 (0.88, 0.97)	**0.92 (0.88, 0.97)**
SMI	1.00 (0.96, 1.05)	0.89 (0.85, 0.93)	**0.89 (0.85, 0.93)**
WIG20	1.00 (0.96, 1.05)	**0.95 (0.91, 1.00)**	0.95 (0.91, 1.00)

**Note**: in parenthesis, the 95% confidence intervals of the non-rejection values of d. In bold the estimated values from the selected model specification.

**Table 9:** Estimated coefficients of the selected models. Daily data. Sample ending on 13 July 2021.

Series (-13/07/2021)	No terms	Constant (t-value)	Time trend (t-value)
i) No autocorrelation			
AEX	0.99 (0.97, 1.02)	5.865 (440.75)	---
BIST100	1.01 (0.99, 1.04)	5.523 (324.60)	---
CAC40	0.96 (0.94, 0.99)	8.259 (578.19)	---
DAX	0.99 (0.97, 1.02)	8.364 (595.96)	0.00037 (1.79)
FTSE-100	0.95 (0.93, 0.98)	8.486 (706.21)	---
FTSE-MIB	0.97 (0.95, 1.00)	10.344 (633.99)	---
IBEX35	0.98 (0.95, 1.01)	9.118 (593.12)	---
OMXS30	0.94 (0.92, 0.97)	6.624 (477.72)	0.00029 (2.09)
SMI	0.98 (0.95, 1.00)	8.661 (766.86)	---
WIG20	1.00 (0.98, 1.03)	7.578 (503.50)	---
ii) With autocorrelation			
AEX	0.98 (0.94, 1.03)	6.006 (429.48)	---
BIST100	1.02 (0.98, 1.07)	5.980 (366.95)	---
CAC40	0.95 (0.91, 1.00)	8.383 (557.17)	---
DAX	0.98 (0.94, 1.03)	8.751 (591.17)	---
FTSE-100	0.94 (0.89, 0.98)	8.617 (692.25)	---
FTSE-MIB	1.00 (0.96, 1.05)	10.238 (577.55)	---
IBEX35	0.95 (0.91, 1.00)	9.348 (572.27)	---
OMXS30	0.92 (0.88, 0.97)	6.759 (478.56)	0.00033 (2.38)
SMI	0.89 (0.85, 0.93)	8.865 (77.16)	0.00018 (2.01)
WIG20	0.95 (0.91, 1.00)	7.829 (523.37)	*---*

**Note**: the values in column 2 are the estimates of d and their 95% confidence bands. In parenthesis, in column 3 and 4, the t-values of the deterministic terms.

**Table 10:** Estimates of the differencing parameter. Weekly data. Sample ending on 13 July 2021.

Series (-13/07/2021)	d (95% band)	Constant	A constant and a linear time trend
i) No autocorrelation			
AEX	1.00 (0.95, 1.05)	**0.99 (0.94, 1.04)**	0.99 (0.94, 1.04)
BIST100	1.00 (0.95, 1.05)	**1.00 (0.96, 1.05)**	1.00 (0.96, 1.05)
CAC40	1.00 (0.95, 1.05)	**0.94 (0.90, 0.99)**	0.94 (0.90, 0.99)
DAX	0.99 (0.95, 1.05)	0.95 (0.90, 1.00)	**0.95 (0.90, 1.00)**
FTSE-100	1.00 (0.95, 1.05)	**0.92 (0.88, 0.98)**	0.92 (0.88, 0.98)
FTSE-MIB	1.00 (0.95, 1.05)	**0.99 (0.95, 1.05)**	0.99 (0.95, 1.05)
IBEX35	0.99 (0.95, 1.05)	**0.95 (0.90, 1.00)**	0.95 (0.90, 1.00)
OMXS30	1.00 (0.95, 1.05)	0.94 (0.90, 1.00)	**0.95 (0.90, 1.00)**
SMI	1.00 (0.95, 1.05)	0.90 (0.86, 0.94)	**0.90 (0.86, 0.94)**
WIG20	1.00 (0.95, 1.05)	**1.00 (0.95, 1.05)**	1.00 (0.95, 1.05)
ii) With autocorrelation			
AEX	0.99 (0.93, 1.09)	**1.01 (0.94, 1.09)**	1.01 (0.94, 1.09)
BIST100	0.99 (0.93, 1.09)	**1.05 (0.96, 1.14)**	1.05 (0.96, 1.14)
CAC40	0.99 (0.92, 1.08)	**0.98 (0.90, 1.08)**	0.98 (0.90, 1.08)
DAX	0.98 (0.92, 1.07)	**0.98 (0.90, 1.09)**	0.98 (0.91, 1.09)
FTSE-100	0.99 (0.92, 1.08)	**0.95 (0.87, 1.05)**	0.96 (0.87, 1.05)
FTSE-MIB	0.99 (0.92, 1.08)	**1.01 (0.94, 1.11)**	1.01 (0.94, 1.11)
IBEX35	0.99 (0.92, 1.08)	**1.00 (0.92, 1.09)**	1.00 (0.92, 1.09)
OMXS30	0.99 (0.93, 1.08)	**0.99 (0.92, 1.07)**	0.99 (0.92, 1.07)
SMI	0.99 (0.92, 1.08)	**1.02 (0.95, 1.10)**	1.02 (0.95, 1.10)
WIG20	0.99 (0.93, 1.08)	**1.00 (0.93, 1.08)**	1.00 (0.93, 1.08)

**Note**: in parenthesis, the 95% confidence intervals of the non-rejection values of d. In bold the estimated values from the selected model specification.

**Table 11:** Estimated coefficients of the selected models. Weekly data. Sample ending on 13 July 2021.

Series (-13/07/2021)	No terms	Constant (t-value)	Time trend (t-value)
i) No autocorrelation			
AEX	0.99 (0.94, 1.04)	5.870 (203.25)	---
BIST100	1.00 (0.96, 1.05)	5.533 (153.50)	---
CAC40	0.94 (0.90, 0.99)	8.264 (277.31)	---
DAX	0.95 (0.90, 1.00)	8.368 (272.56)	0.00148 (1.94)
FTSE-100	0.92 (0.88, 0.98)	8.488 (342.71)	---
FTSE-MIB	0.99 (0.95, 1.05)	10.345 (306.50)	---
IBEX35	0.95 (0.90, 1.00)	9.110 (281.41)	---
OMXS30	0.95 (0.90, 1.00)	6.616 (236.07)	0.00128 (1.84)
SMI	0.90 (0.86, 0.94)	8.655 (257.25)	0.00079 (1.78)
WIG20	1.00 (0.95, 1.05)	7.551 (239.92)	---
ii) With autocorrelation			
AEX	1.01 (0.94, 1.09)	5.869 (203.28)	---
BIST100	1.05 (0.96, 1.14)	5.530 (153.84)	---
CAC40	0.98 (0.90, 1.08)	8.263 (276.64)	---
DAX	0.98 (0.90, 1.09)	8.370 (272.42)	---
FTSE-100	0.95 (0.87, 1.05)	8.488 (341.19)	---
FTSE-MIB	1.01 (0.94, 1.11)	10.345 (306.52)	---
IBEX35	1.00 (0.92, 1.09)	9.109 (281.07)	---
OMXS30	0.99 (0.92, 1.07)	6.617 (235.94)	---
SMI	1.02 (0.95, 1.10)	8.654 (356.67)	---
WIG20	1.00 (0.93, 1.08)	7.551 (239.92)	---

**Note**: the values in column 2 are the estimates of d and their 95% confidence bands. In parenthesis, in column 3 and 4, the t-values of the deterministic terms.

**Table 12:** Estimates of the differencing parameter. Monthly data. Sample ending on 13 July 2021.

Series (-13/07/2021)	d (95% band)	Constant	A constant and a linear time trend
i) No autocorrelation			
AEX	0.99 (0.91, 1.11)	**1.09 (1.00, 1.21)**	1.09 (1.00, 1.21)
BIST100	0.98 (0.89, 1.09)	0.97 (0.86, 1.11)	**0.97 (0.87, 1.11)**
CAC40	0.99 (0.90, 1.10)	**1.04 (0.95, 1.17)**	1.04 (0.95, 1.17)
DAX	0.99 (0.90, 1.10)	1.01 (0.90, 1.16)	**1.01 (0.91, 1.16)**
FTSE-100	0.99 (0.90, 1.10)	**0.99 (0.89, 1.11)**	0.99 (0.89, 1.11)
FTSE-MIB	0.99 (0.90, 1.10)	**1.02 (0.93, 1.14)**	1.02 (0.93, 1.14)
IBEX35	0.99 (0.90, 1.10)	**1.01 (0.91, 1.13)**	1.01 (0.91, 1.13)
OMXS30	0.99 (0.91, 1.11)	**1.04 (0.95, 1.15)**	1.04 (0.95, 1.15)
SMI	0.99 (0.90, 1.10)	**1.12 (1.02, 1.24)**	1.12 (1.02, 1.23)
WIG20	1.00 (0.91, 1.11)	**1.03 (0.94, 1.15)**	1.03 (0.94, 1.15)
ii) With autocorrelation			
AEX	0.98 (0.83, 1.15)	**1.01 (0.85, 1.23)**	1.01 (0.85, 1.23)
BIST100	0.94 (0.81, 1.12)	0.89 (0.65, 1.16)	**0.88 (0.67, 1.16)**
CAC40	0.97 (0.83, 1.17)	**0.91 (0.75, 1.12)**	0.92 (0.76, 1.12)
DAX	0.96 (0.83, 1.16)	0.80 (0.63, 1.02)	**0.80 (0.63, 1.02)**
FTSE-100	0.96 (0.82, 1.16)	**0.91 (0.73, 1.16)**	0.91 (0.73, 1.16)
FTSE-MIB	0.96 (0.83, 1.16)	**0.94 (0.79, 1.16)**	0.94 (0.79, 1.16)
IBEX35	0.94 (0.83, 1.15)	**0.89 (0.72, 1.12)**	0.89 (0.71, 1.12)
OMXS30	0.98 (0.83, 1.16)	**1.06 (0.89, 1.31)**	1.06 (0.87, 1.30)
SMI	0.97 (0.82, 1.17)	**1.06 (0.88, 1.30)**	1.06 (0.88, 1.29)
WIG20	0.97 (0.83, 1.17)	**1.00 (0.80, 1.22)**	1.00 (0.79, 1.22)

**Note**: in parenthesis, the 95% confidence intervals of the non-rejection values of d. In bold the estimated values from the selected model specification.

**Table 13:** Estimated coefficients of the selected models. Monthly data. Sample ending on 13 July 2021.

Series (-13/07/2021)	No terms	Constant (t-value)	Time trend (t-value)
i) No autocorrelation			
AEX	1.09 (1.00, 1.21)	5.883 (120.86)	---
BIST100	0.97 (0.87, 1.11)	5.602 (74.06)	0.0080 (1.73)
CAC40	1.04 (0.95, 1.17)	8-270 (168.50)	---
DAX	1.01 (0.91, 1.16)	8.348 (157.08)	0.0066 (1.68)
FTSE-100	0.99 (0.89, 1.11)	8.587 (216.74)	---
FTSE-MIB	1.02 (0.93, 1.14)	10.352 (168.10)	---
IBEX35	1.01 (0.91, 1.13)	9.129 (158.50)	---
OMXS30	1.04 (0.95, 1.15)	6.605 (141.51)	---
SMI	1.12 (1.02, 1.24)	8.655 (246.68)	---
WIG20	1.03 (0.94, 1.15)	7.539 (122.31)	---
ii) With autocorrelation			
AEX	1.01 (0.85, 1.23)	5.886 (120.34)	---
BIST100	0.88 (0.67, 1.16)	5.604 (74.92)	0.889 (2.54)
CAC40	0.91 (0.75, 1.12)	8.277 (170.42)	---
DAX	0.80 (0.63, 1.02)	8.362 (165.24)	0.800 (4.39)
FTSE-100	0.91 (0.73, 1.16)	8.492 (218.81)	---
FTSE-MIB	0.94 (0.79, 1.16)	103.53 (168.98)	---
IBEX35	0.89 (0.72, 1.12)	9.135 (161.04)	---
OMXS30	1.06 (0.89, 1.31)	6.604 (141.69)	---
SMI	1.06 (0.88, 1.30)	8.657 (245.12)	---
WIG20	1.00 (0.80, 1.22)	7.542 (122.34)	---

**Note**: the values in column 2 are the estimates of d and their 95% confidence bands. In parenthesis, in column 3 and 4, the t-values of the deterministic terms.

In the case of the daily series a time trend is required for DAX and OMXS-30 without autocorrelation and OMXS-30 and SMI with autocorrelation (exactly the same as for the shorter sample), and mean reversion is found in the case of FTSE-100 and OMXS-30 with white noise errors and these two series along with SMI with autocorrelated disturbances. As for the weekly series, DAX, OMXS-30 and SMI require a time trend with white noise errors, and under the same assumption CAC-40, FTSE-100 and SMI display a small degree of mean reversion. Finally, of the monthly series BIST-100 and DAX are the only two with a significantly positive time trend with both white noise and autocorrelated errors, and the unit root null hypothesis cannot be rejected in any single case except for SMI with white noise errors, in this case in favour of d > 1.

Tables 14 and 15 display a summary of the results discussed above. In brief, under the assumption of white noise errors, there is a slight increase in the degree of integration at the daily and weekly frequency but a decrease at the monthly one. In the more realistic case of autocorrelated disturbances a different picture emerges: in general, the degree of persistence decreases, especially at the weekly and monthly frequencies. However, the differences are not statistically significant, which suggests that the Covid-19 pandemic has had very little effect on the degree of persistence of stock markets.

**Table 14:**
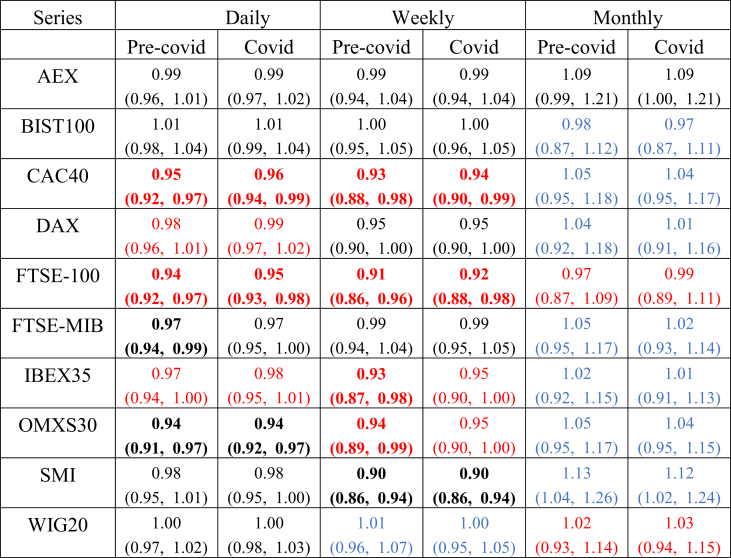
Summary of the Results. Comparison of the values of d in a model with white noise errors. **Note:** in bold, cases of mean reversion. Red (blue) indicates an increase (decrease) in the estimated degree of persistence when using the full sample.

**Table 15:**
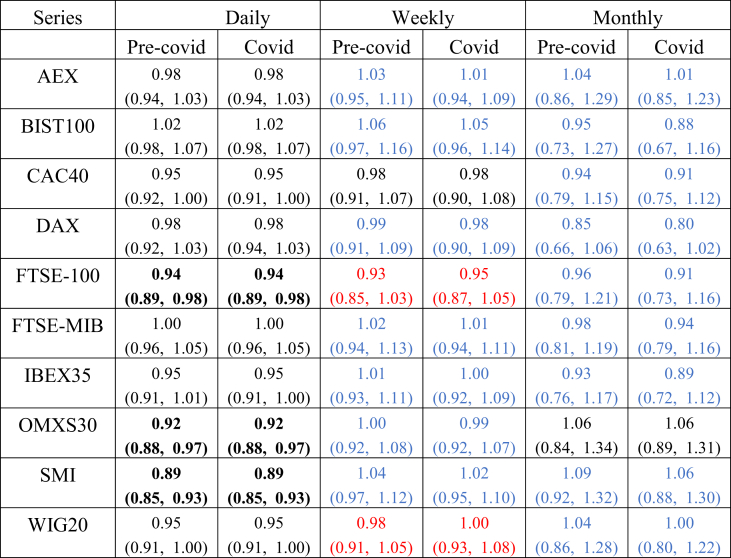
Summary of the Results. Comparison of the values of d in a model with autocorrelated errors. **Note:** in bold, cases of mean reversion. Red (blue) indicates an increase (decrease) in the estimated degree of persistence when using the full sample.

Finally we also analyse persistence in the squared returns of the series under examination, which is a proxy for volatility. More specifically, we estimate again the parameter d at all three frequencies for both the pre-Covid sample ending on 31 December 2019, and the full sample ending ending on 13 July 2021, and compare the respective estimates to assess the impact of the pandemic. These results are displayed in Table 16.

**Table 16:**
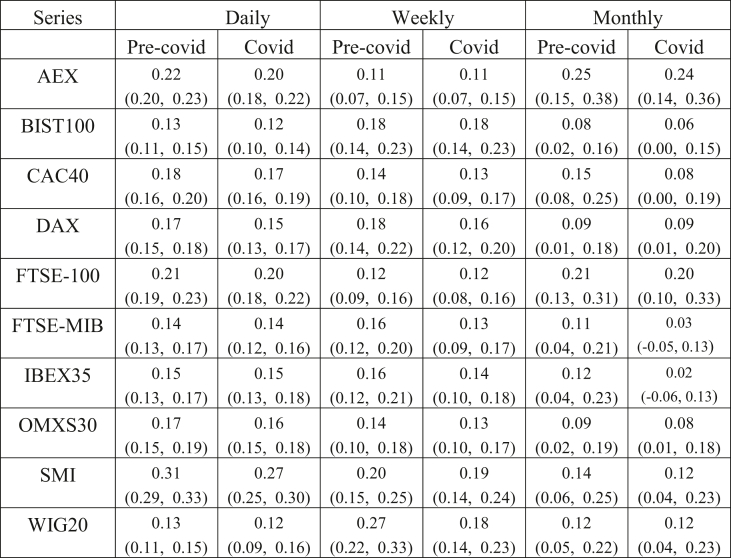
Estimates of d on the squared returns series. **Note:** in bold, cases of mean reversion. Red (blue) indicates an increase (decrease) in the estimated degree of persistence when using the full sample.

In general, the value of d is found to decrease for the full sample including the Covid-19 period, especially in the case of SMI at the daily frequency (from 0.31 to 0.27), WIG20 at the weekly frequency (from 0.27 to 0.18) and CAC40 at the monthly frequency (from 0.15 to 0.08). Evidence of long memory is found in all cases examined and similar results are obtained using absolute returns instead of the squared ones. These findings suggest that the increase in uncertainty caused by the pandemic is not having long-lasting effects on the volatility of stock prices and that this will relatively quickly revert to pre-Covid levels.

The results presented above are not directly comparable to those obtained using more restrictive approaches imposing the I(0) versus I(1) dichotomy on the series of interest (see, e.g., Shehzad et al., 2020; Al-Awadhi et al., 2020; Corbet et al., 2020; Albulescu, 2020; etc.), but only to others also based on fractional integration. However, most of them focus on the US rather than Europe and use a multivariate framework allowing for the possible effects of various epidemiological and health factors on stock markets (see, e.g., Abakah et al., 2021, 2022; Caporale et al., 2022c), which again makes a direct comparison inappropriate. The closest study to the present one is Caporale et al. (2022a), which examines persistence in US bond yields as well as stock prices. Interestingly, their recursive analysis suggests that the Covid-19 pandemic did not affect the persistence of stock prices, which is consistent with our findings for the European stock markets. It would appear, therefore, that the impact of the recent health crisis on financial markets was not as pronounced as that of previous events such as the Global Financial Crisis of 2007–2008.

## 5 Conclusions

This paper analyses the impact of the Covid-19 pandemic on the degree of persistence of European stock markets. Specifically, it uses fractional integration methods to estimate persistence at the daily, weekly and monthly frequencies in the case of ten major European stock market indices; the effects of the pandemic are assessed by comparing the pre-pandemic estimates (over the period 2005–2019) to those from a sample extended until July 2021 which includes the pandemic period. The approach used is more general than the standard one based on the stationarity versus non-stationarity dichotomy and allows for a wider range of dynamic processes. Three different model specifications are considered, and these are estimated under two alternative assumptions for the disturbances (white noise and autocorrelation). The findings indicate that there has not been any significant impact of the Covid-19 pandemic on the degree of persistence of European stock market indices, which is in line with the results reported by Caporale et al. (2022b) for the US concerning the rather limited impact of the recent health crisis on financial markets. In fact the volatility process appears to have become less persistent, which suggests that the higher uncertainty faced by investors during the pandemic is not going to have long-lasting effects.

A limitation of the analysis carried out in this paper is that it does not allow for nonlinear structures. In fact, testing structural breaks or nonlinearities is a very important issue in this context since fractional integration could be spuriously generated by breaks which are not taken into account. Our focus is on the impact of the Covid-19 pandemic on the degree of persistence of the series of interest; however, the period analysed also includes other possible breaks such as the 2007/08 financial crisis which will be investigated in future work.

## Declarations

### Author contribution statement

Guglielmo Maria Caporale; Luis Gil-Alana; Isabel Arrese Lasaosa: Conceived and designed the experiments; Performed the experiments; Analyzed and interpreted the data; Contributed reagents, materials, analysis tools or data; Wrote the paper.

### Funding statement

This research did not receive any specific grant from funding agencies in the public, commercial, or not-for-profit sectors.

### Data availability statement

Data will be made available on request.

### Declaration of interest's statement

The authors declare no conflict of interest.

### Additional information

No additional information is available for this paper.

## Footnotes

1 See Diebold and Rudebusch (1989), Sowell (1992a) and Gil-Alana and Robinson (1997) for applications involving *I(d)* processes in macroeconomic time series.

2 More precisely, the sample period goes from 4 January 2005 to 13 July 2021 for the daily and monthly series, and from 7 January 2005 to 9 July 2021 for the weekly ones.
